# Endothelial cells metabolically regulate breast cancer invasion toward a microvessel

**DOI:** 10.1063/5.0171109

**Published:** 2023-12-04

**Authors:** Matthew L. Tan, Niaa Jenkins-Johnston, Sarah Huang, Brittany Schutrum, Sandra Vadhin, Abhinav Adhikari, Rebecca M. Williams, Warren R. Zipfel, Jan Lammerding, Jeffrey D. Varner, Claudia Fischbach

**Affiliations:** 1 Nancy E. and Peter C. Meinig School of Biomedical Engineering, Cornell University, Ithaca, New York 14853, USA; 2 Robert Frederick Smith School of Chemical and Biomolecular Engineering, Cornell University, Ithaca, New York 14853, USA; 3 Kavli Institute at Cornell for Nanoscale Science, Cornell University, Ithaca, New York 14853, USA

## Abstract

Breast cancer metastasis is initiated by invasion of tumor cells into the collagen type I-rich stroma to reach adjacent blood vessels. Prior work has identified that metabolic plasticity is a key requirement of tumor cell invasion into collagen. However, it remains largely unclear how blood vessels affect this relationship. Here, we developed a microfluidic platform to analyze how tumor cells invade collagen in the presence and absence of a microvascular channel. We demonstrate that endothelial cells secrete pro-migratory factors that direct tumor cell invasion toward the microvessel. Analysis of tumor cell metabolism using metabolic imaging, metabolomics, and computational flux balance analysis revealed that these changes are accompanied by increased rates of glycolysis and oxygen consumption caused by broad alterations of glucose metabolism. Indeed, restricting glucose availability decreased endothelial cell-induced tumor cell invasion. Our results suggest that endothelial cells promote tumor invasion into the stroma due, in part, to reprogramming tumor cell metabolism.

## INTRODUCTION

Breast cancer mortality is driven by metastasis, the process by which cells leave the primary tumor to develop secondary tumors at distant sites in the body.^1^ Metastasis is a multi-step process that is initiated by local cell invasion into the surrounding stroma.^2^ The extent of invasion is determined by the distinct biophysical and biochemical properties of the stromal microenvironment.^3^ One critical component that determines these properties is the extracellular matrix (ECM), a network of proteins, carbohydrates, and other molecules with which cells interact via adhesion receptors including integrins.^4^ Additionally, growth factors and cytokines secreted by stromal cells regulate cancer invasion in concert with the ECM microenvironment.^5–7^ However, most invasion studies focus on only one of these parameters, while *in vivo* ECM and secreted factors act on cells simultaneously, a process that can induce synergistic effects. Studying tumor cells in the context of relevant matrix and biochemical cues will, therefore, improve understanding of the mechanisms underlying tumor invasion.

Collagen type I is the most abundant matrix component in the tumor stroma. Indeed, relative to healthy tissue, the stroma of tumors and other conditions known to promote tumorigenesis is characterized by increased collagen deposition and remodeling.^8–10^ Increased collagen density, in turn, can facilitate cancer invasion and metastasis via altered adhesion formation and signaling.^4,11,12^ Another key feature of the stroma are blood vessels that tumor cells explore in order to travel to distant metastatic sites.^13^ However, the mechanisms that tumor cells use to navigate the collagen-rich stroma to reach vessels are poorly understood. While previous research has revealed that tumor cells attract blood vessels by secreting proangiogenic factors, it is now clear that the opposite may occur as well. For example, studies in conventional monolayer culture or mouse models found that endothelial cells (ECs) secrete signaling molecules that can influence tumor cell behavior.^14–16^ How ECs influence tumor cell invasion in the context of a dense 3D collagen matrix remains unclear.

One mechanism by which tumor cells regulate their invasive properties is by reprogramming their metabolism. Even in the presence of oxygen, cancer cells preferentially convert glucose to lactate to produce adenosine triphosphate (ATP) via aerobic glycolysis. This process termed the Warburg effect is thought to supply precursors that fuel tumor survival and biomass generation and is often associated with dysregulated signaling pathways.^17–21^ As an energy intensive process, 3D invasion through dense matrices requires tumor cells to increase ATP production and subsequent glucose consumption.^22,23^ How the presence of endothelial cells affects the metabolism of invasive tumor cells and which pathway-specific alterations are involved in this process remain unclear.

To elucidate how ECs affect tumor cell invasion into the ECM and which role metabolic reprogramming plays in this process, convergent approaches are needed that allow studying tumor cell invasion experimentally while probing the underlying metabolic changes comprehensively with computational approaches. Microfluidic platforms are especially attractive experimental models of the tumor–vasculature interface as they enable both the formation of functional vascular channels within a 3D matrix and the analysis of tumor cell phenotype in a spatiotemporally controlled manner.^24,25^ Indeed, microfluidic models have been used to study how tumor cell–EC interactions affect angiogenesis, drug response, and tumor cell intra- and extravasation.^14^ These platforms could be combined with flux balance analysis (FBA) models, which draw on metabolomics data to simulate a desired cell phenotype and computationally predict the resulting metabolic fluxes in a genome-scale network of cell metabolism.^26^ FBA modeling enables comprehensive metabolic analysis beyond metabolite measurements and has been previously used to study tumor metabolism.^27,28^ Nevertheless, FBA models have not yet been explored to elucidate the metabolic regulation of tumor cell invasion in response to a functional vasculature component.

In this study, we have developed a microfluidic collagen type I hydrogel that incorporates two parallel channels of which one is seeded with tumor cells and the other is coated with endothelial cells. Subsequently, we used this model to study tumor cell invasion toward the endothelial channel and to measure the metabolic phenotype of 3D invading tumor cells in real-time using fluorescence lifetime imaging (FLIM) of NADH autofluorescence. Similar studies were also performed using conditioned media (CM) to determine the role of EC-secreted factors in regulating invasion and metabolism. Further metabolic analysis was performed by Seahorse analysis and metabolomics. Results from these studies were subsequently used to perform FBA for the identification of specific metabolic pathways that may be involved in EC-mediated tumor cell invasion. Finally, we demonstrate the relevance of our findings by restricting glucose levels or adding metabolic inhibitors to investigate the functional consequences of EC-mediated metabolic reprogramming in tumor cell invasion.

## RESULTS

### A microfluidic device for 3D co-culture of tumor and endothelial cells

**FIG. 1.**
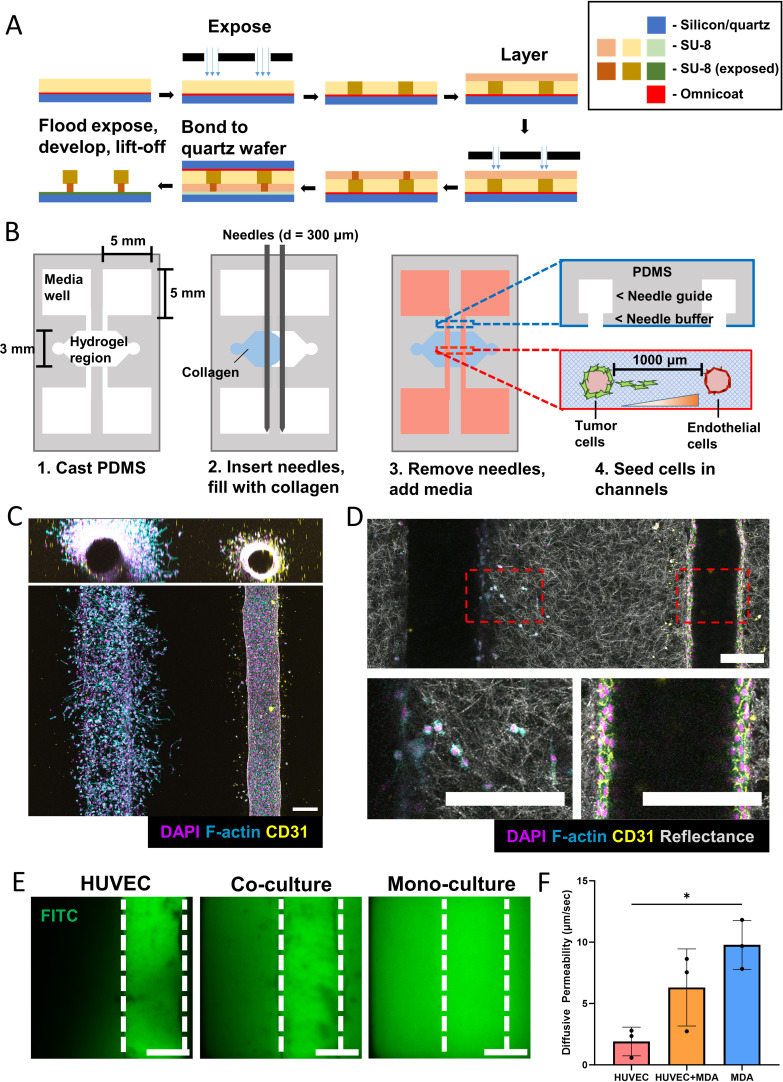
3D microfluidic device to study tumor invasion in response to endothelial cells. (a) Schematic demonstrating the triple layer fabrication method starting with top–down layer deposition, followed by transfer to a new wafer in the correct orientation. (b) Schematic for the generation of microfluidic devices. (c) Representative maximum intensity projection (MIP) of a confocal tile scan of microfluidic device, demonstrating the formation of a confluent endothelial cell channel with a hollow lumen and a tumor cell channel invading into the surrounding fibrillar collagen gel (scale bar = 200 *μ*m). (d) Confocal reflectance projection demonstrating fibrillar structure of collagen hydrogel (scale bar = 200 *μ*m). (e) Confocal slice of fluorescein isothiocyanate (FITC) diffusing from a channel without and with HUVECs (scale bar = 150 *μ*m). (f) Resulting diffusive permeability calculations for a HUVEC channel cultured alone (HUVEC), a HUVEC channel cultured alongside a MDA-MB-231 channel (HUVEC + MDA), and an empty collagen channel cultured alongside a MDA-MB-231 channel (MDA) (n = 3 biological replicates, error bars represent standard deviation). *p < 0.05.

To study how endothelial cells affect 3D tumor cell invasion, we engineered a collagen-based microfluidic co-culture platform containing a tumor and microvascular compartment. First, device molds were fabricated using a reverse, triple layer SU-8 photolithography method by adapting a previously established protocol, where SU-8 layers are deposited top–down onto a sacrificial layer before being transferred to a new wafer in the correct orientation, forming an overhang structure that enables a suspended channel [Fig. 1(a)].^29^ Next, microfluidic devices were created using a needle pull-through method^24^ on a microfabricated polydimethylsiloxane (PDMS) chip to form two hollow cylindrical channels fully embedded in a type I collagen hydrogel [Fig. 1(b)]. The chip is adhered to a glass coverslip to enable confocal imaging. To initiate cell culture in devices, human umbilical vein endothelial cells (HUVECs) were first seeded into one channel of the device, where they formed a confluent monolayer with functional cell–cell adhesions as validated by CD31 staining [Figs. 1(c) and 1(d)]. Subsequently, MDA-MB-231 were seeded in the opposite channel and invaded into the collagen hydrogel with a directional bias toward the EC channel [Fig. 1(c)]. This migration was not mediated by fabrication-dependent changes of collagen fiber alignment as confocal reflectance imaging confirmed that the hydrogel formed between both channels consisted of randomly oriented fibers [Fig. 1(d)]. Analysis of fluorescein isothiocyanate (FITC) diffusion revealed that microvessels in the presence of tumor cells were more permeable than control vessels consistent with the fact that tumor-associated vessels are more leaky than healthy vessels.^30^ More specifically, ECs in a monoculture context provided a significant barrier to FITC diffusion relative to an empty collagen channel [Figs. 1(e) and 1(f)] while ECs cultured with tumor cells displayed intermediate permeability. Collectively, these results suggest that the microfluidic platform developed here enables 3D co-culture of tumor cells and ECs within collagen, and that these two cell types can communicate with each other via soluble factors.

### Endothelial cells induce invasion and morphological changes in breast cancer cells

**FIG. 2.**
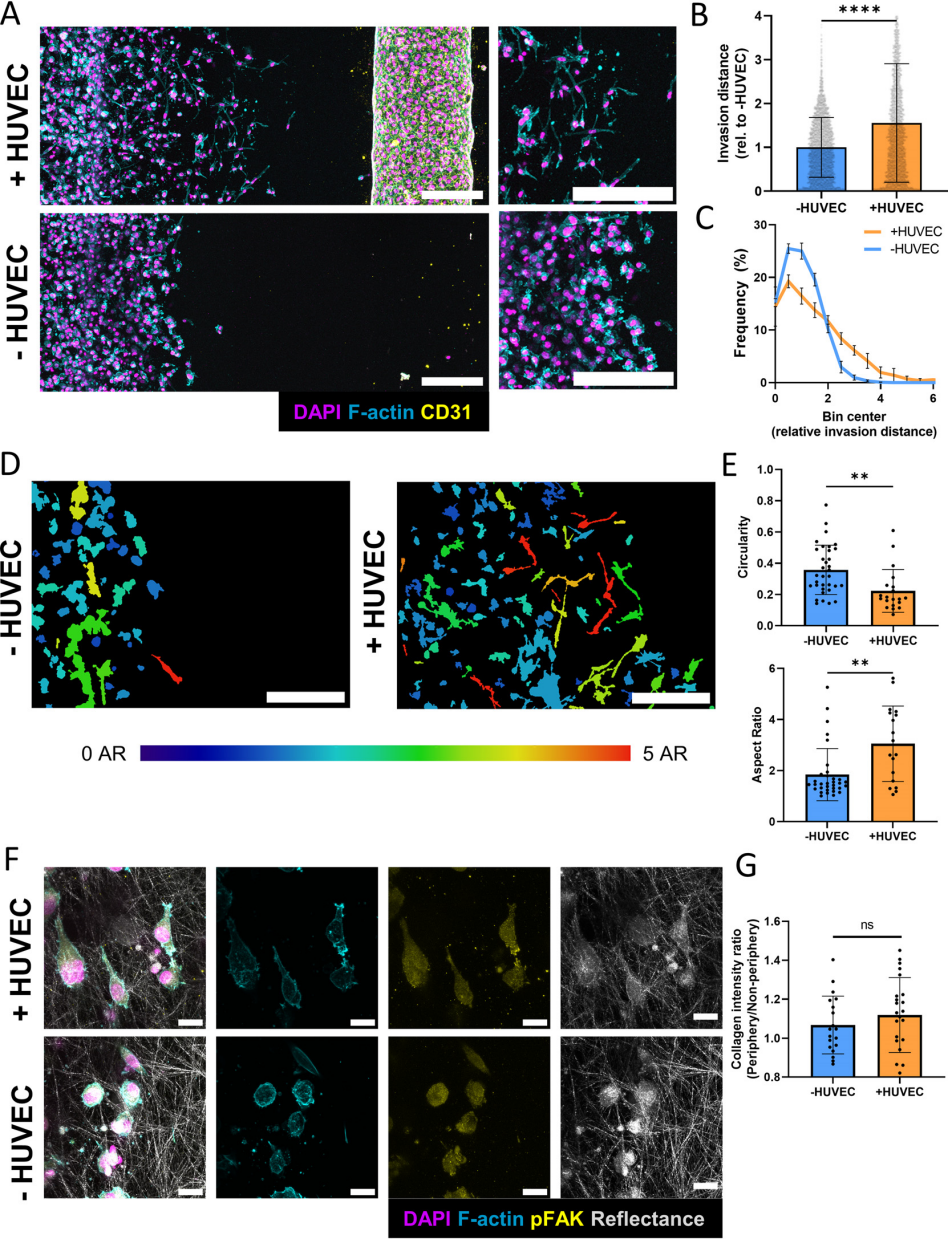
Endothelial cells increase cancer cell invasion and induce morphological changes. (a) Representative fluorescent confocal projections of tumor cells invading toward an EC-coated or empty channel (scale bar = 200 *μ*m). (b) Quantification of invasion distance in microfluidic devices in either co-culture (+HUVEC) or tumor cell only (−HUVEC) conditions (n = 3 biological replicates, data points represent individual cells, error bars represent standard deviation). (c) Distribution of invasion distances in microfluidic devices (n = 3 biological replicates, data points represent individual cells, error bars represent standard deviation). (d) Representative image of cell shapes within microfluidic device pseudo-colored according to aspect ratio (AR). The left side of the images represent the edge of the tumor channel (scale bar = 200 *μ*m). (e) Quantification of morphological characteristics of tumor cells within microfluidic devices (n = 3 biological replicates, data points represent individual cells, error bars represent standard deviation). (f) Representative confocal projections of tumor cells at the invasive front in microfluidic devices stained for F-actin and pFAK (scale bar = 20 *μ*m). (g) Ratio of collagen intensity adjacent to cells relative to bulk collagen intensity in microfluidic devices (n = 3 devices, data points represent separate field of views (FOVs), n < 5 FOV per device). ^*^p < 0.05, ^**^p < 0.01, ^****^p < 0.0001.

To investigate the effect of ECs on tumor cell invasion in more detail, MDA-MB-231 were cultured in the microfluidic device in the presence or absence of an EC-coated channel [Fig. 2(a)]. When cultured together with ECs, tumors cells invaded significantly more relative to the monoculture condition [Figs. 2(b) and 2(c)]. Additionally, the presence of ECs caused MDA-MB-231 to assume more elongated morphologies, with lower circularity and higher aspect ratio (AR) consistent with a more invasive phenotype [Figs. 2(d) and 2(e)]. Interestingly, these morphological changes correlated with distance from the EC channel, where the most elongated cells were localized closest to the ECs [Fig. 2(d)]. Because changes of cell shape and invasion depend on altered adhesion characteristics,^31^ MDA-MB-231 were assessed for levels of phosphorylated focal adhesion kinase (pFAK), a protein that is upregulated in invading cells and metastasis^32^ [Fig. 2(f)]. Despite the detected morphological and invasive differences pFAK levels in MDA-MB-231 were similar in both conditions and presented as diffuse signals in the cytoplasm rather than distinct focal adhesions [Fig. 2(f)]. These findings are not unexpected as invasion and adhesion complex formation of tumor cells in 3D is regulated differently than in 2D cell culture and depends on the formation of pseudopodial protrusions and deformation of the surrounding matrix.^33^ Although cell elongation in 3D matrices depends on increased protrusion formation,^34^ transcellular collagen density indicative of matrix contraction did not vary statistically between both conditions [Figs. 2(f) and 2(g)]. Together, these results indicate that ECs promote invasion in breast cancer cells, and that this increased invasion is associated with differences in cell morphology at the invasion front.

### Endothelial cell-secreted factors promote cancer cell migration in 2D and 3D

**FIG. 3.**
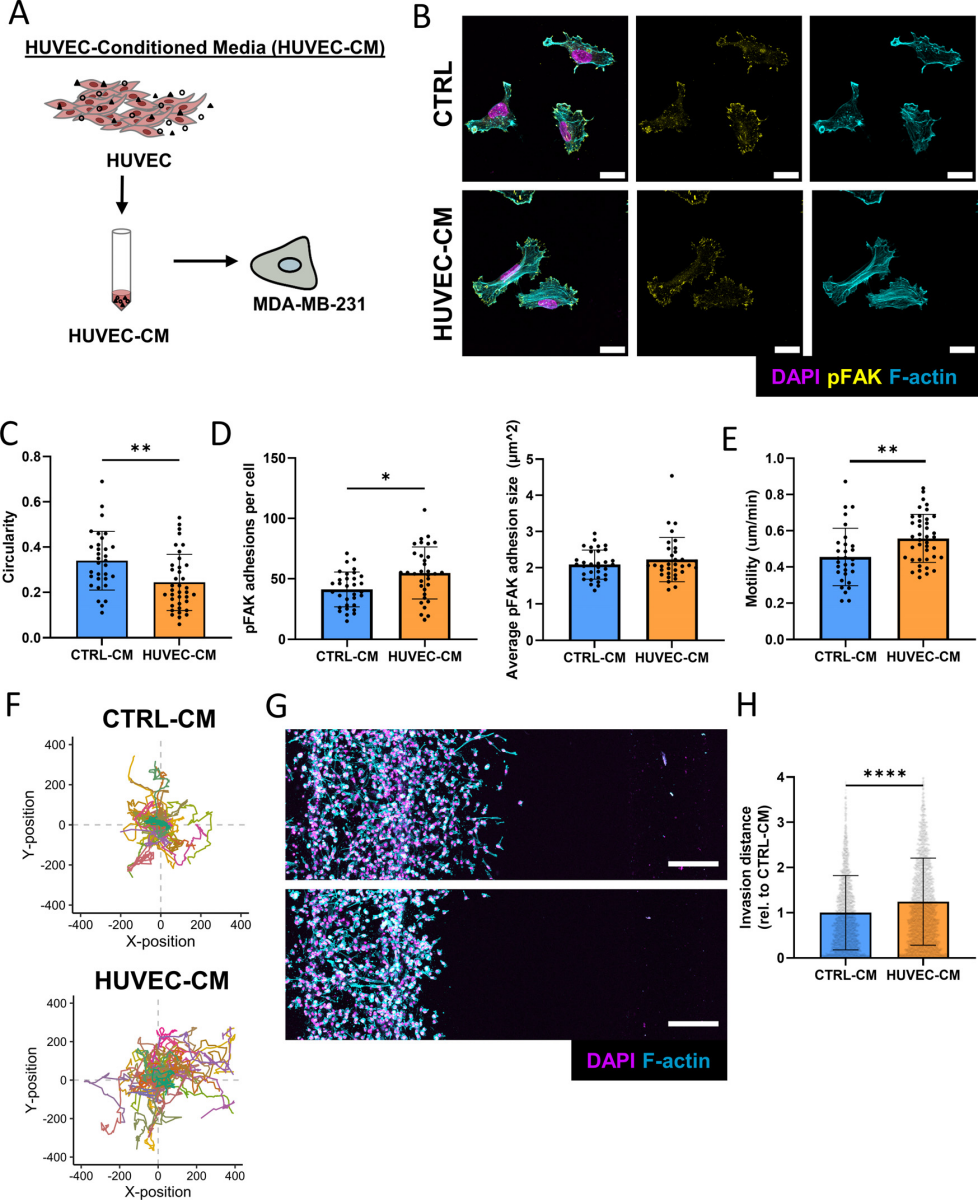
Endothelial cell secreted factors induce an invasive phenotype. (a) Schematic for the generation of HUVEC-CM. (b) Representative confocal images of immunostained MDA-MB-231 treated with or without HUVEC-CM (scale bar = 20 *μ*m). (c) Circularity was measured from cells treated with or without HUVEC-CM (n = 3 biological replicates, data points represent individual cells, error bar represents standard deviation). (d) Average number and size of pFAK adhesions per cell of MDA-MB-231 treated with or without HUVEC-CM (n = 3 biological replicates, data points represent individual cells, error bar represents standard deviation). (e) Motility of cells treated with CTRL-CM or HUVEC-CM as analyzed using the Incucyte live cell imaging platform (n > 40 cells per condition, data points represent individual cells, error bar represents standard deviation). (f) Migration plot of MDA-MB-231 treated with CTRL-CM or HUVEC-CM (n > 40 cells per condition). (g) Representative fluorescent confocal projections depicting invasion of MDA-MB-231 when treated with HUVEC-CM (top) or CTRL-CM (bottom) (scale bar = 200 *μ*m). (h) Corresponding quantification of invasion distances of MDA-MB-231 (n = 3 biological replicates, data points represent individual cells, error bar represents standard deviation). ^*^p < 0.05, ^**^p < 0.01, ^****^p < 0.0001.

Because increased tumor cell invasion in response to ECs can be observed without direct cell–cell contact, we speculated that factors secreted by ECs were responsible for the detected differences. To test this, we collected concentrated conditioned media from HUVECs (HUVEC-CM) and subsequently diluted it with fresh media so that the final concentration of HUVEC secreted proteins was twofold relative to the original concentration [Fig. 3(a)]. Subsequently, we tested the effect of HUVEC-CM or control media that was not exposed to HUVECs but processed identically (CTRL-CM) on MDA-MB-231 adhesion and migration in both conventional 2D culture and our microfluidic devices. Interestingly, 2D-cultured MDA-MB-231 treated with HUVEC-CM were more elongated (as indicated by a decrease in circularity) and, distinct from 3D, formed more, but similarly sized pFAK positive adhesions per cell relative to cells treated with CTRL-CM [Figs. 3(b)–3(d)]. Additionally, incubation with HUVEC-CM increased the average motility and overall migration distance of MDA-MB-231 in 2D culture, corroborating the increase in invasion seen in 3D [Figs. 3(e) and 3(f)]. This result was further supported using a transwell migration assay, where the presence of ECs in the lower compartment increased migration of MDA-MB-231 through the porous membrane [Fig. S1(a)]. To determine the effect of HUVEC-secreted factors on invasion in 3D, tumor cells were seeded in microfluidic devices while the opposite channel, which is otherwise seeded with ECs, was used to supply CTRL-CM or HUVEC-CM. Indeed, HUVEC-CM was sufficient to increase MDA-MB-231 morphological changes and invasion, confirming that EC-secreted factors contributed to our findings in the co-culture platform [Figs. 3(g) and 3(h)]. These findings were not due to differences in proliferation as MDA-MB-231 treated with HUVEC-CM grew less and contained fewer Ki67 positive cells relative to cells treated with CTRL-CM [Figs. S1(b) and S1(c)]. These results demonstrate that EC-secreted factors stimulate migration and invasion of breast cancer cells in both 2D and 3D contexts.

### Endothelial cell-secreted factors induce metabolic changes in tumor cells

**FIG. 4.**
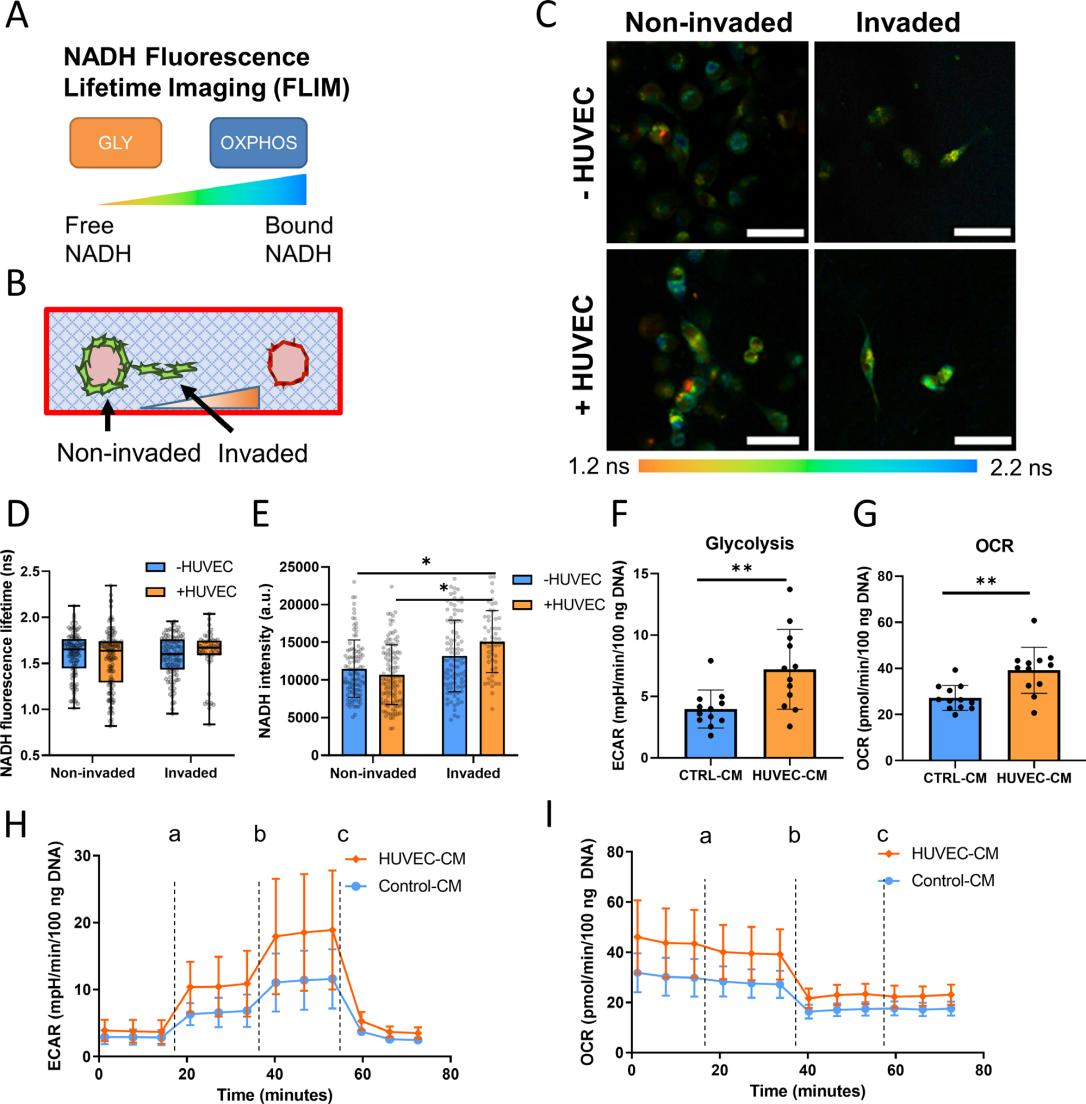
Endothelial cell secreted factors induce increased energy metabolism in tumor cells. (a) Schematic demonstrating fluorescence lifetime imaging (FLIM) of NADH to analyze metabolic phenotype. (b) Schematic of NADH FLIM strategy in devices to differentiate between invaded and non-invaded cells. (c) Representative NADH FLIM images in microfluidic devices along with (d) NADH fluorescence lifetime values and (e) NADH intensity values in MDA-MB-231 (n = 3 biological replicates, data points represent individual cells). Error bars represent minimum and maximum for (d), and standard deviation for (e) (scale bar = 50 *μ*m). (f) Seahorse metabolic flux analysis of extracellular acidification rate (ECAR) and (g) oxygen consumption rate (OCR) to represent glycolysis and oxidative metabolism, respectively (n = 12 wells, error bars represent standard deviation). (h) Seahorse ECAR and (i) OCR traces for the glycolysis stress test, a—glucose addition, b—oligomycin addition, c—2-DG addition (n = 12 wells, error bars represent standard deviation). ^*^p < 0.05, ^**^p < 0.01.

As tumor cell invasion is closely tied to cellular metabolism, we sought to characterize the metabolic phenotype of invading tumor cells exposed to EC-secreted factors. This was accomplished using NADH fluorescence lifetime imaging (FLIM), a label-free metabolic imaging technique to assess cancer cell metabolic state.^35,36^ By observing the lifetime of NADH autofluorescence, it is possible to determine if a cell is more glycolytic (free NADH with a lower lifetime) or more oxidative (bound NADH with a longer lifetime)^37^ [Fig. 4(a)]. This technique is especially well-suited to determine the metabolic states of invaded and non-invaded tumor cells in the microfluidic device as it allows for spatially resolved analysis *in situ* [Fig. 4(b)]. Interestingly, the average NADH lifetime of MDA-MB-231 was independent of the presence of ECs and did not vary between invaded and non-invaded cells [Figs. 4(c) and 4(d)]. However, NADH intensity, which is indicative of NADH concentration within a cell,^37^ was increased when MDA-MB-231 invaded in the presence of ECs [Fig. 4(e)]. Consistent with this result, real-time metabolic analysis with Agilent Seahorse analyzer using the glycolysis stress test kit revealed that MDA-MB-231 treated with HUVEC-CM increased both their extracellular acidification rate (ECAR) and oxygen consumption rate (OCR), indicating an increase in both glycolysis and oxidative metabolism, respectively [Figs. 4(f)–4(i)]. Taken together, these results indicate that EC-secreted factors stimulate metabolic changes in tumor cells that correlate with increased invasiveness.

### Convergent approaches reveal HUVEC-CM induces broad metabolic alterations in tumor cells

**FIG. 5.**
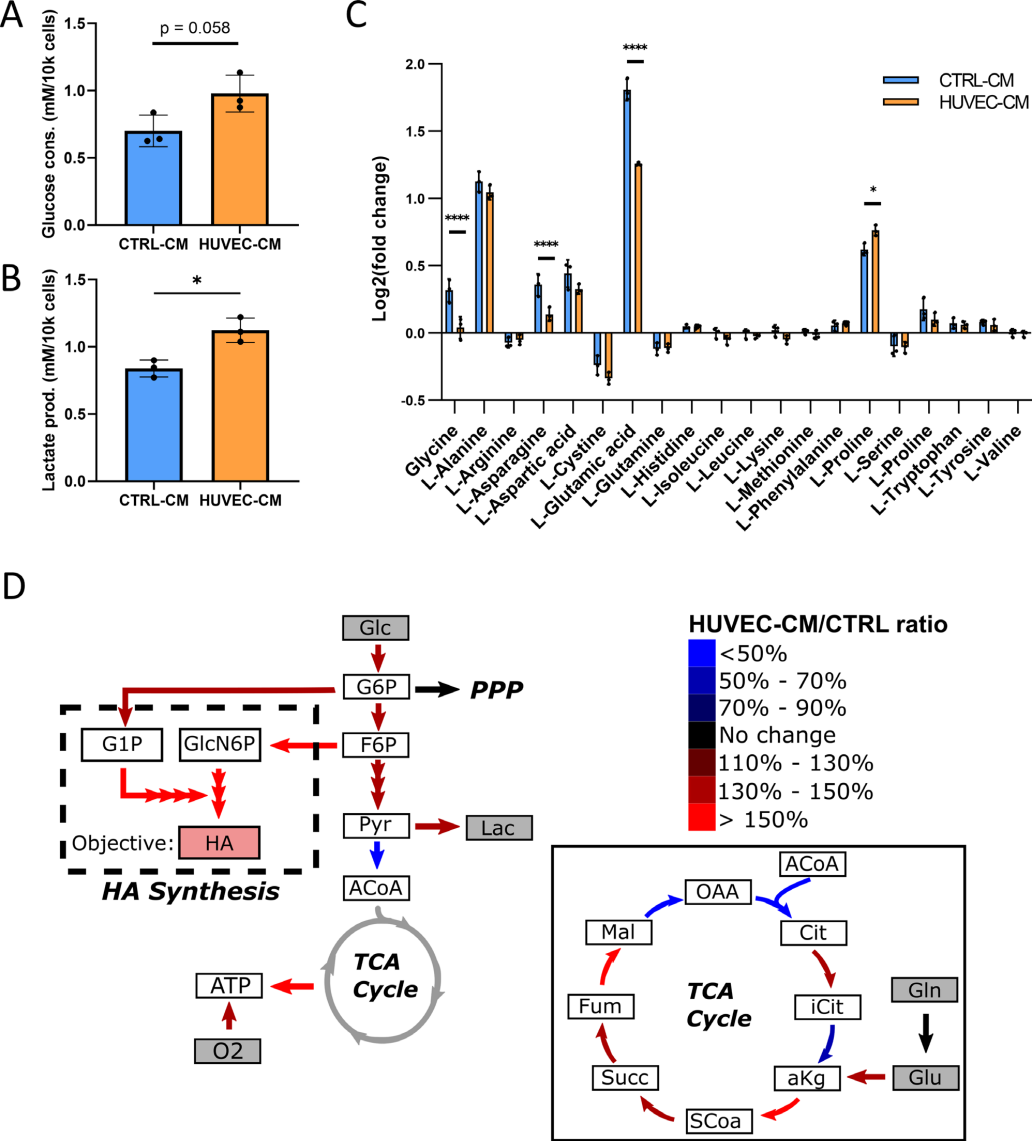
Metabolomic analysis of tumor cells treated with endothelial cell secreted factors indicate broader metabolic alterations. (a) Glucose consumption of MDA-MB-231 treated with or without HUVEC-CM for 48 h (n = 3 wells, error bar represents standard deviation). (b) Lactate production of MDA-MB-231 treated with or without HUVEC-CM for 48 h (n = 3 wells, error bar represents standard deviation). (c) Log twofold changes of the 20 amino acids of MDA-MB-231 treated with or without HUVEC-CM for 48 h relative to starting amounts at t = 0 h (n = 3 wells, error bar represents standard deviation). (d) Graphical representation of a subset of FBA model results pertaining to the bioenergetic pathways of MDA-MB-231 treated with or without HUVEC-CM for 48 h. Inset contains expanded FBA results for the TCA cycle. Metabolites highlighted in gray represent those that were measured experimentally and used to constrain FBA results. A full list of the FBA model constraints and simulated reactions can be found in supplementary material File 1. ^*^p < 0.05, ^****^p < 0.0001.

As our Seahorse data suggested that exposure to HUVEC-CM increased glycolysis in tumor cells [Figs. 4(f) and 4(h)], we directly measured glucose consumption and lactate production in cells treated with HUVEC-CM or CTRL-CM. While not statistically significant, MDA-MB-231 treated with HUVEC-CM trended toward increased consumption of glucose relative to control conditions [Fig. 5(a)]. Accordingly, treatment with HUVEC-CM increased lactate production and thus, glycolytic metabolism by tumor cells [Fig. 5(b)]. To probe alterations in additional metabolic pathways, extracellular metabolomics was performed to measure the secretion/uptake profiles of the canonical amino acids when MDA-MB-231 were treated with or without HUVEC-CM for 48 h. When exposed to HUVEC-CM, MDA-MB-231 secreted less glycine, L-asparagine, and L-glutamic acid (glutamate) relative to cells that were treated with CTRL-CM [Fig. 5(c)]. Additionally, MDA-MB-231 treated with HUVEC-CM secreted more proline compared to those treated with CTRL-CM [Fig. 5(c)]. Glucose, lactate, and the amino acid secretion/consumption profiles were subsequently used to constrain a flux balance analysis (FBA) model to computationally predict changes in specific metabolic pathways using genome-scale reconstructions of metabolic networks [Fig. 5(d)].^26^ This enables us to simulate and probe alterations in metabolic fluxes by tuning model parameters such as the objective function that maximizes a specific reaction and flux constraints. Using this approach, we demonstrated previously that overproduction of hyaluronic acid (HA) by cancer stem-like cells increases their invasion into a 3D collagen matrix due to elevated glycolytic metabolism.^38^ As EC-secreted factors also increased tumor cell invasion and glycolytic metabolism, we speculated that changes in tumor cell stemness may underlie these observations. Interestingly, exposure of MDA-MB-231 reporter cells that express green fluorescent protein (GFP) under control of the stem cell transcription factor NANOG (GFP-NANOG) to HUVEC-CM increased the fraction of GFP^high^, stem-like cells relative to treatment with CTRL-CM [Figs. S2(a) and S2(b)]. Additionally, co-culturing hyaluronic acid synthase 2 (HAS2) overexpressing, more stem-like MCF10A breast epithelial cells (HAS2-MCF10A) with HUVECs increased their migration through a transwell, while co-culturing estrogen receptor-positive MCF7 breast cancer cells with HUVECs did not show a significant difference in migration [Figs. S2(c) and S2(d)]. Because these results suggest a possible connection between EC-induced invasion with stemness and HA production, we utilized our previous FBA method and set the objective function to maximize HA production.^38^ Results from this analysis indicate that exposure to HUVEC-CM enables tumor cells to increase their production of HA and ATP [Fig. 5(d)]. Furthermore, an increase in the upper-stages of glycolysis, conversion of pyruvate to lactate, and the conversion of glutamate to α-ketoglutarate into the tricarboxylic acid (TCA) cycle was observed. These findings could help explain the increase in glycolysis, glucose consumption, and lactate production that we measured experimentally [Figs. 4(f), 5(a), and 5(b)] and justify the increased NADH autofluorescence detected by FLIM [Fig. 4(e)] as the generation and oxidation of α-ketoglutarate are known to produce NADH.^39,40^ By combining experiments with computational FBA, we cross-validated both methods, while elucidating potential alterations in genome scale bioenergetic pathways responsible for our experimental results.

### Glucose restriction decreases tumor cell invasion in response to EC-secreted factors to a greater degree than ATP synthase inhibition

**FIG. 6.**
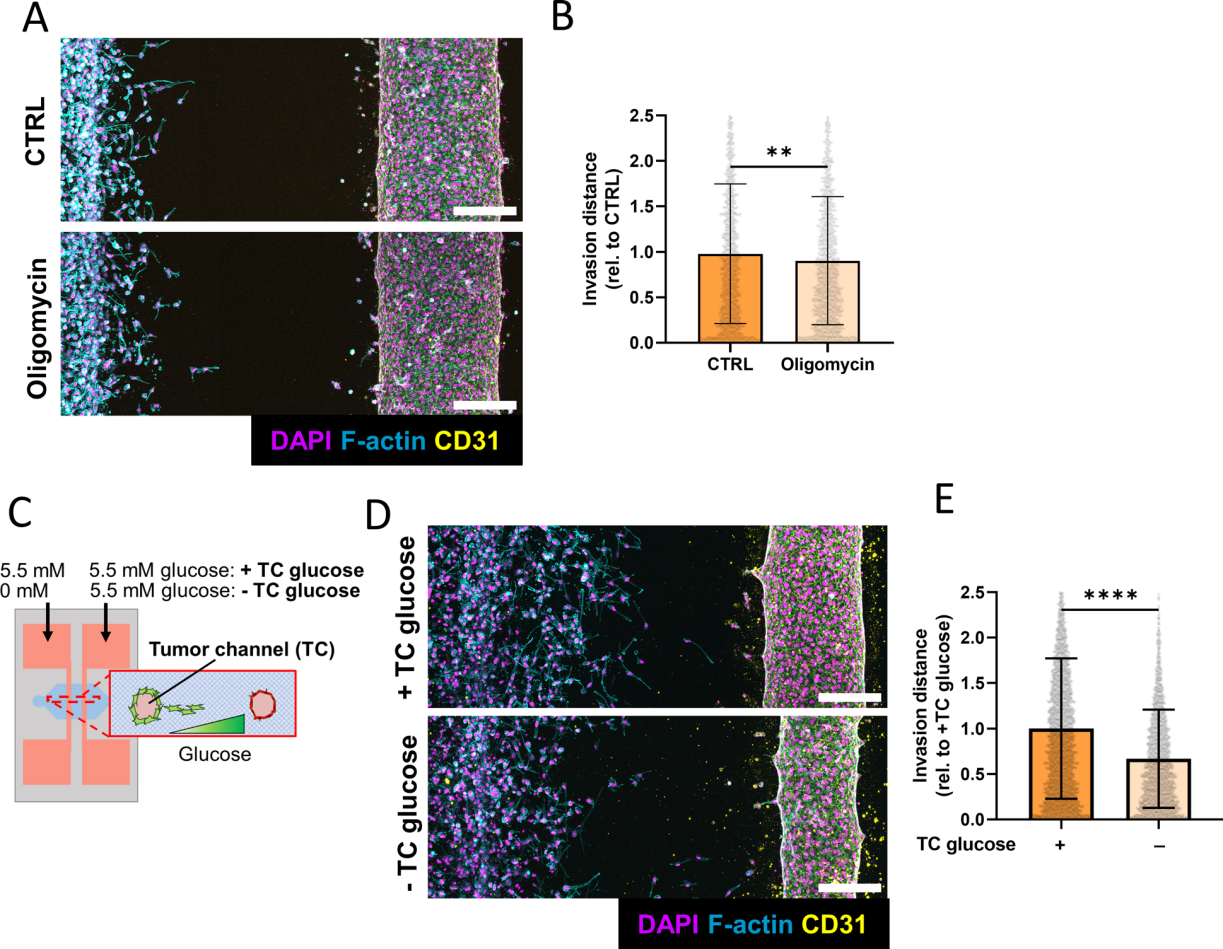
Glucose restriction and ATP synthase inhibition inhibit endothelial cell-dependent tumor cell invasion. (a) Confocal projection of MDA-MB-231 invading toward an EC channel with or without oligomycin treatment for 24 h (scale bar = 200 *μ*m). (b) Quantification of invasion distance in microfluidic devices with or without oligomycin treatment (n = 3 biological replicates, data points represent individual cells, error bars represent standard deviation). (c) Schematic demonstrating glucose restriction in microfluidic devices. (d) Confocal projection of MDA-MB-231 invading toward an EC channel with or without glucose restriction for 5 days (scale bar = 200 *μ*m). (e) Quantification of invasion distance in microfluidic devices with or without glucose restriction (n = 3 biological replicates, data points represent individual cells, error bars represent standard deviation). ^**^p < 0.01, ^****^p < 0.0001.

As tumor cells treated with EC-secreted factors exhibited increased NADH levels, glycolysis, and oxidative metabolism (Figs. 4 and 5), and FBA predicted an increase in ATP production [Fig. 5(d)], we speculated that increased invasion could be linked to an increase in ATP production. To test this, co-culture devices were treated for 24 h with oligomycin (longer incubation times were avoided due to the cytotoxicity of the drug), a potent ATP synthase inhibitor that can reduce metastatic potential in tumor cells.^41^ For these experiments, oligomycin was added to both channels to prevent effects caused by the formation of inhibitor gradients. Interestingly, the addition of oligomycin, although statistically significant, only mildly inhibited tumor cell invasion in the presence of ECs [Figs. 6(a) and 6(b)] but had no effect when added to devices lacking ECs [Figs. S3(a) and S3(b)]. As we have previously shown that increased ATP production could be a result of increased glycolytic metabolism and that glycolytic metabolism is necessary for invasion, we next investigated the effects of glucose restriction.^38^ To accomplish this, glucose-free media was supplied to the tumor channel, while EGM-2 containing 5.5 mM glucose was used to feed the EC channel. This approach effectively reduced the amount of glucose in the tumor region of the device, while preventing HUVEC death [Fig. 6(c)]. Depriving the tumor channel of glucose significantly decreased MDA-MB-231 invasion both in the presence and absence of ECs, but this effect was more pronounced in response to ECs [Figs. 6(d), 6(e), S3(c), and S3(d)]. Collectively, these results suggest that ECs promote tumor invasion by increasing glycolytic metabolism and that more efficient ATP production may play a role in this process.

## DISCUSSION AND CONCLUSIONS

To study how ECs affect tumor invasion into the collagen-rich stroma and determine which role metabolic changes play in this process, we have developed a collagen type 1-based microfluidic platform consisting of a tumor cell-seeded channel and an adjacent HUVEC-coated microvessel. While most previous studies focused on how tumor cells affect ECs, we now demonstrate that EC-secreted factors stimulate 3D tumor cell invasion. NADH FLIM and Seahorse-based analysis revealed that tumor cells exposed to EC-secreted factors upregulate energy producing pathways, including glycolysis, and oxygen consumption. Metabolic inhibition experiments further supported that these metabolic changes contributed to EC-mediated tumor invasion. This was complemented by extracellular metabolomics to measure the secretion and consumption profile of glycolytic metabolites and the canonical amino acids to constrain an FBA model, indicating EC-secreted factors induce broad metabolic alterations beyond glycolytic pathways. In particular, EC-secreted factors appeared to increase the production of lactate in glycolysis and the generation of NADH in the TCA cycle, two distinct mechanisms that lead to increased ATP production. Therefore, EC-secreted factors may induce increased invasion through an abundance of ATP, which has previously been shown as a requirement for invasion through dense matrices.^22,23^

Microfluidic model systems have advanced our understanding of tumor invasion, intravasation, and extravasation.^42,43^ Nevertheless, current platforms are often limited in their ability to study tumor–EC interactions in relevant 3D microenvironmental contexts. For example, 2D microfluidic models have yielded important new insights into tumor cell migration,^44^ but 3D invasion is known to be distinctly regulated from 2D migration.^31^ Additionally, fibrin-based microfluidic models are often used to stimulate robust vascular responses for subsequent studies,^45,46^ but this matrix does not faithfully recapitulate ECM in the tumor stroma which is predominantly collagen-based. Finally, microfluidic models are attractive tools to study tumor metabolism in response to metabolic gradients in a 3D microenvironment;^47,48^ however, prior systems did not include invasive tumor cells and lacked an integrated vascular channel or network. The model developed in this work addresses some of these shortcomings by embedding cells in a 3D fibrous collagen matrix, while enabling the study of tumor invasion and metabolism in response to naturally generated metabolic and cell signaling gradients.

While it is widely accepted that the cellular composition of the tumor microenvironment plays a significant role in the metabolic regulation of cancer, most previous work has focused on elucidating the functional contributions of cell types other than ECs on tumor cell metabolism, including cancer associated fibroblasts, immune cells such as macrophages, and mesenchymal stem cells.^49,50^ Indeed, only a handful of studies have shown that EC-mediated signaling causes metabolic modulation in tumor cells. For instance, ECs were found to induce increased glycan and lipid metabolism in cancer cells.^51^ More recently, cancer cells cultured together with ECs exhibited decreased phosphoglycerate dehydrogenase (PHGDH) expression, potentiating metastatic dissemination.^52^ Interestingly, glycan metabolism and PHGDH activity have direct or indirect effects on glycolysis,^52,53^ which we show here increases in tumor cells that have been exposed to EC-secreted factors. Although our FBA model indicates an increased capacity for HA production, which is directly linked to glycan metabolism, whether or not these pathways are modulated in our system necessitates further experimentation for validation, including the direct measurement of HA production by tumor cells treated with EC-secreted factors using enzyme-linked immunosorbent assays (ELISA) or immunofluorescent staining.

Although it has been shown by others that ECs can promote tumor cell migration or metabolic reprogramming, the related experiments were all done in conventional monolayer culture and did not establish a functional link between both observations. For example, ECs were found to stimulate tumor cell migration by inducing a stem-like phenotype, but how metabolic changes affected this process was not examined.^15,54,55^ A connection likely exists as the stem-like phenotype in tumor cells is metabolically regulated and characterized by increased glycolysis and oxidative metabolism.^38,56,57^ Moreover, ECs secrete factors that promote tumor cell stemness, for example by activating interleukin-6 (IL-6)/Notch and transforming growth factor beta (TGF-β) signaling.^16,58,59^ In our hands, treatment with HUVEC-CM increased the fraction of stem-like tumor cells [Figs. S2(a)–S2(b)] and stimulated migration of more stem-like, HA-overproducing tumor cells relative to control cells [Fig. S2(c)]. When maximizing HA production in our FBA model, we also found that HUVEC-CM treated tumor cells increased upper glycolysis and oxidative metabolism similar to our previous work in which increased HA production promoted cancer stem-like cell (CSC) invasion by stimulating ATP biosynthesis.^38^ As HA contributes to the acquisition of a stem-like phenotype^57^ and vascular invasion,^43,60^ the EC-dependent increase in tumor cell invasion and energy metabolism detected in our work could be related to the induction of a stem-like tumor cell phenotype and corresponding HA production. To investigate this possibility and identify the underlying molecular mechanism, future experiments will need to test how specific EC-secreted factors, including IL-6 or TGF-β, affect tumor cell expression of stem cell and metabolic genes in our experimental setting and perturb the related signaling pathways with gene ablation or function blocking studies. Concurrent studies will need to relate findings to differences in metabolism and HA production.

Differences in adhesion dynamics and cytoskeletal rearrangement are determinants of cell morphology and have been connected to cell migration and invasion.^61,62^ Furthermore, cell morphology in 2D culture can predict how tumor cells invade 3D substrates under similar biochemical conditions; however, the mechanisms driving tumor cell migration vary between 2D and 3D culture settings.^31,33^ Consistent with these previous findings, our data suggests that EC-secreted factors induce tumor cell migration and more elongated and protrusive cell morphologies both in 2D and 3D cultures. Yet tumor cells in 2D cultures formed adhesion complexes with distinct pFAK foci that increased in quantity in the presence of EC-secreted factors while the pFAK staining intensity or pattern in 3D-cultured tumor cells did not change. This is perhaps not surprising, as cells embedded in 3D have been shown previously to lack distinct focal adhesion complexes. Instead, focal adhesion proteins in 3D embedded cells modulate cell motility through protrusion activity and matrix deformation.^33,63^ An alternative explanation is that many other signaling molecules that were not considered here are involved in adhesion formation and can be differentially regulated in 2D and 3D. For example, matrix remodeling through the secretion of matrix metalloproteinases (MMP)^64^ or collagen-mediated discoidin domain receptor (DDR) signaling^11^ have been shown to promote tumor invasion independent of focal adhesion formation.^61^ To better understand how EC-secreted regulate adhesion formation in 2D vs 3D culture and how these changes influence matrix remodeling and tumor cell invasion further studies including *in situ* imaging of collagen fiber structure and the use of various inhibitors that interfere with adhesion complex formation are necessary.

Due to the difficulty of performing Seahorse flux analysis and metabolomics in 3D co-cultures, we utilized 2D cultures treated with HUVEC-CM for detailed metabolic analysis and FBA modeling. Extracellular metabolomics revealed alterations in metabolites other than those implicated directly in glycolysis. Glutamic acid/glutamate secretion was significantly reduced in MDA-MB-231 treated with HUVEC-CM relative to the control. Glutamate is an intermediate in the conversion of glutamine to α-ketoglutarate, one of the substrates in the TCA cycle. Glutamine metabolism has been implicated in tumor malignancy, where increased glutamine consumption can lead to deregulated energetics along with increased proliferation and invasion.^20^ However, glutamate can also act independently of glutamine, where excess extracellular amounts can bind to glutamate receptors and activate signaling pathways to promote proliferation, survival, and migration.^65^ This conflicts with our results, where EC-secreted factors increased invasion of tumor cells, as well as decreased glutamate secretion. Additionally, as glutamine consumption was not increased in cells treated with HUVEC-CM, decreased glutamate secretion could indicate an increase in glutamate consumption and thus α-ketoglutarate production. Indeed, our FBA model predicted an increase in glutamate conversion to α-ketoglutarate and subsequently to succinyl-CoA. In addition to the conversion of glutamate to α-ketoglutarate, the oxidation of α-ketoglutarate itself has been implicated in increased NADH generation in cancer cells, providing a potential explanation for the increased NADH signal seen in our FLIM studies.^66^ However, additional metabolic studies will need to be performed to confirm this hypothesis. Additionally, as ECs may behave differently in 3D relative to 2D, future metabolic analysis should include HUVEC-CM harvested from ECs cultured in a 3D matrix.

Although the platform used here provides a robust model to study tumor invasion toward the vasculature, future experiments with alternative vascular and tumor cell types would further advance the broader conclusions from our work. HUVECs are derived from venous and neonatal sources and thus may not mimic the specific phenotype of ECs present in the tumor microenvironment. Moreover, relative to healthy ECs, tumor-associated ECs secrete more angiocrine factors while giving rise to leaky and disorganized vasculature.^55,67^ Perivascular cells such as vascular smooth muscle cells and pericytes can normalize vascular permeability and function^68^ but can also establish a pro-metastatic environment.^69^ Collectively, these changes in EC secretion and vascular transport may modulate tumor invasion *in vivo*, a possibility that could be studied by introducing human or murine tumor-derived ECs in combination with pericytes into the device.^70,71^ Another aspect to consider is that most of our experiments used MDA-MB-231 breast cancer cells as a model of triple negative, highly invasive breast cancer, but different breast cancer subtypes may elicit varied responses. Indeed, estrogen receptor-positive MCF7 breast cancer cells appeared to migrate more in response to HUVEC-CM, but these differences were not statistically significant [Fig. S2(d)]. To determine the broader relevance of our findings, future studies testing additional cell lines or patient-derived organoids from different cancer subtypes in our microfluidic model are warranted. Collectively, such studies could also help explain why vascular channels were more permeable in the presence of tumor cells but did not exhibit other signs of vascular dysfunction as was shown previously with similar models of tumor/vascular interactions.^72^

## METHODS

### Cell culture

MDA-MB-231 and MCF7 (ATCC) cells were cultured in high glucose Dulbecco's Modified Eagle Medium (DMEM) supplemented with 10% fetal bovine serum (FBS) and 1% penicillin/streptomycin. GFP-NANOG MDA-MB-231 (a gift from Dr. Ofer Reizes^73^) cells were cultured in DMEM supplemented with 10% FBS and 1% penicillin/streptomycin. Human umbilical vein endothelial cells (HUVEC) (Lonza) were routinely cultured in EGM-2 (Lonza) and used under passage 4. HAS2-MCF10A were cultured in DMEM/F12 supplemented with 5% horse serum, 1% penicillin/streptomycin, 10 *μ*g/ml insulin, 0.5 *μ*g/ml hydrocortisone, 100 ng/ml cholera toxin, and 20 ng/ml human epidermal growth factor (EGF).

### Fabrication of silicon master mold for soft lithography

Device molds were fabricated using a reverse, triple layer SU-8 photolithography method by adapting previously established protocols.^24,29,74^ Briefly, layers were sequentially deposited and cured on a silicon wafer from the bottom up, starting with the media/gel layer, followed by the needle guide layer, and finally the needle buffer layer [Figs. 1(a) and S4]. Silicon wafers were first cleaned with piranha solution and dehydrated on a temperature-controlled hotplate at 150 °C for 15 min. 5 ml of Omnicoat (Kayaku) was deposited to the center of the wafer and spun at 500 RPM at 100 RPM/s for 5 s, followed by 3000 RPM at 300 RPM/s for 30 s. Coated wafers were baked at 200 °C for 1 min and allowed to cooldown to RT.

For the media/gel layer, SU-8 2100 was deposited and spun at 500 RPM at 100 RPM/s for 10 s, followed by 1500 RPM at 300 RPM/s for 30 s for a 200 *μ*m thick layer. Wafers were soft baked at 55 °C overnight. UV exposure occurred at 350 mJ/cm^2^. Post exposure bake occurred at 55 °C for 2 h. For the needle guide layer, SU-8 2150 was deposited and spun at 500 RPM at 100 RPM/s for 10 s, followed by 1950 RPM at 300 RPM/s for 60 s to form a 300 *μ*m layer. A soft bake occurred at 55 °C overnight. 400 mJ/cm^2^ of UV was applied using the contact aligner (ABM). Post exposure bake occurred at 55 °C for 2 h, and the wafer was allowed to cool before proceeding to the next layer. SU-8 2100 was deposited onto the wafer and spun at 500 RPM at 100 RPM/s for 10 s, followed by 3000 RPM at 300 RPM/s for 30 s for a 100 *μ*m thick layer. A soft bake was performed overnight at 55 °C. The coated wafer was exposed to 300 mJ/cm^2^ of UV using a contact aligner (ABM). Post exposure bake occurred at 55 °C for 2 h and the wafer was allowed to cool completely before depositing the next layer. Wafers were allowed to cool completely before development in SU-8 developer for 1.5 h.

After development, a second, float-glass wafer was coated with a 10 *μ*m layer of SU-8. The silicon wafer containing device features was bonded to this second wafer using a SB8e substrate bonder (SUSS MicroTec). After bonding, the sandwiched wafer was exposed to 300 mJ/cm^2^ UV to complete adhesion to the second wafer. To separate the patterns from the silicon wafer, the sandwich was developed in MF-319 (Microposit) until the sandwich separated.

### Fabrication of microfluidic devices and collagen scaffolds

To generate molds for microfluidic devices, a polydimethylsiloxane (PDMS) (Dow) solution consisting of elastomer and curing agent at a ratio of 1:10 was degassed and poured into the silicon master to cure overnight in an oven set at 60 °C. After removal from the oven and cooling, PDMS devices were removed from the master mold and inlets/media wells were cut out. PDMS molds were then cleaned using O_2_ plasma for 5 min before bonding to plasma-cleaned #1 cover glasses (Chemglass). Assembled devices were then treated with 1% polyethylenimine and 0.1% glutaraldehyde for 10 and 30 min, respectively, before washing with de-ionized sterile water overnight. The next day, devices were dried and UV-sterilized for 10 min before inserting 300 *μ*m diameter acupuncture needles (Seiren) that were previously coated with 1% bovine serum albumin (BSA) in phosphate-buffered saline (PBS) overnight and subsequently UV-sterilized for another 10 min. A 2.5 mg/ml solution of rat tail collagen type I (Corning) was adjusted to neutral pH using 1 N NaOH, injected into devices, and polymerized at 4 °C for 30 min, followed by polymerization at 37 °C for 30 min. Needles were carefully removed, and devices were rinsed with EGM-2 for 24 h before subsequent cell seeding.

### Cell culture in microfluidic devices

To seed ECs in devices, media in both channels were replaced with fresh EGM-2. A suspension of 2 × 10^6^ HUVECs per mL EGM-2 was subsequently prepared. 10 *μ*l of this suspension was injected into the right (EC) channel and devices were flipped upside down and incubated at 37 °C for 10 min. Subsequently, a second seeding was performed after flipping devices right-side up, and devices were incubated at 37 °C for another 10 min. Media in cell-seeded devices was replaced with fresh EGM-2, and devices were incubated at 37 °C on a rocker set at 15° tilt angle and 2 RPM for 48 h before tumor cell seeding.

To seed tumor cells, a suspension of 2 × 10^6^ MDA-MB-231 per mL was prepared in EGM-2. Next, 10 *μ*l of this suspension was injected into one port of the left (tumor) channel, followed by injection of another 10 *μ*l into the opposite port of the left (tumor) channel. Cells were allowed to attach for 30 min before changing media. Unless otherwise stated, high glucose DMEM supplemented with 1% FBS, 1% P/S (low serum DMEM) was added to the tumor channel, while fully supplemented EGM-2 (which contains 2% FBS) was added to the EC channel. Devices were placed back on the rocker in the incubator and cultured for 3–5 days. Media was replaced every 24 h.

To investigate the effects of glucose deprivation in microfluidic devices, media in the tumor channel was changed to either low glucose (5.5 mM) low serum DMEM to match glucose levels in EGM-2 or glucose-free low serum DMEM to simulate glucose deprivation. Devices were cultured for 5 days with daily media changes before fixation and subsequent staining and imaging.

To investigate the effects of ATP synthesis inhibition, 2 *μ*m of oligomycin A was added to both channels of the device 24 h after tumor cell seeding. Devices were treated for 24 h before fixation and subsequent staining and imaging. Longer incubation times were avoided due to cytotoxic effects of the treatment after prolonged incubation times.

### Analysis of endothelial permeability

To characterize the functionality of the EC-coated channel via analysis of diffusive permeability, media was removed from all reservoirs. Subsequently, fresh EGM-2 was added to the tumor cell channel while a 10 *μ*M solution of fluorescein isothiocyanate (FITC) (Sigma) in EGM-2 was added to the EC channel. Immediately, the devices were imaged on an Andor/Olympus spinning disk confocal using a 10×/0.4 USPLSAPO10 × 2 objective. Images were acquired every 2 s for 5 min.

Diffusive permeability was calculated as described for a similar device elsewhere.^24^ Briefly, following Fick's First Law, the diffusive permeability can be described as

$$J=P_{d}\left(c_{\text{vessel}}-c_{\mathrm{ECM}}\right),$$ (1)

where c_vessel_ is the concentration of fluorescein in the vessel and c_ECM_ is the concentration of FITC in the surrounding matrix. J, defined as the flux of fluorescein into the matrix, is given as follows:

$$J=\frac{dn_{\mathrm{ECM}}}{dt}*\frac{1}{A},$$ (2)

where n is the number of solute molecules, t is time, and A is the cross-sectional area of the channel. Assuming the intensity of fluorescence is proportional to the number of molecules, the channel is cylindrical, the optical thickness is much less than the radius of the vessel (which is true for confocal imaging), and that the concentration of solute is greater in the vessel than the matrix initially, then the diffusive permeability is

$$P_{D}=(\frac{2r_{\text{vessel}}}{I_{\text{vessel}}})(\frac{dI_{\mathrm{ECM}}}{dt}),$$ (3)

where r_vessel_ is the radius of the channel, I_vessel_ is the initial intensity in the channel, and dI_ECM_/dt is the slope of the linear portion of the change in integrated density of fluorescence in the matrix adjacent to the vessel.

### Experiments with HUVEC-conditioned media (HUVEC-CM)

To generate HUVEC-CM, a confluent T-150 of HUVECs was cultured in reduced EGM-2 lacking the exogenous growth factors VEGF, hFGF, R3-IGF, and hEGF for 24 h. Conditioned media was collected and centrifuged at 3220 × g to remove floating cells and debris, and subsequently frozen at −20 °C. Prior to use, conditioned media was normalized to 7 × 10^6^ cells, concentrated 10× using a centrifuge concentration filter with a molecular weight cutoff of 3 kDa (Millipore) and reconstituted in fresh low serum DMEM to a final concentration of 2×. For control conditions, reduced EGM-2 was processed identically. HUVEC-CM and control-CM (CTRL-CM) were used within one week and stored at 4 °C between uses.

To assess the effect of HUVEC-CM on tumor cell adhesion, MDA-MB-231 were seeded at 1000 cells per well onto 96-well glass bottom plates coated with 50 *μ*g/ml rat tail collagen type I. Cultures were treated with HUVEC-CM or CTRL-CM for 48–72 h before fixing and subsequent staining and image analysis. To assess the effect of EC-secreted factors on random migration, MDA-MB-231 cells were plated on collagen-coated (50 *μ*g/ml) 96 well glass bottom plates and placed in an Incucyte S3 (Sartorius) live cell imaging system in either HUVEC-CM or CTRL-CM. Images were obtained in 20-min intervals over 24 h. Individual cell tracking was performed using ImageJ to determine migration velocity (motility) and random migration paths. To determine the effect of HUVEC-CM on tumor cell invasion in microfluidic cultures, tumor cells were seeded and cultured in the left channel as described above, while the right channel that is otherwise seeded with ECs was used to supply either HUVEC-CM or CTRL-CM. Devices were cultured as usual for 5 days.

To measure the effects of HUVEC-CM on cancer cell stemness, GFP-NANOG MDA-MB-231 were treated with HUVEC-CM or CTRL-CM for 48 h. Cells were trypsinized and resuspended at 10 × 10^6^ cells/ml in flow sorting buffer (2.5% FBS/PBS, 2 mM EDTA) before being analyzed on a BD Accuri C6 Plus Analyzer (BD Biosciences).

### Real-time metabolic analysis

Real-time changes in metabolism were tested using a Seahorse XFe96 Analyzer in conjunction with the Seahorse XF Glycolysis Stress Test Assay Kit (Agilent). 2D cultures of MDA-MB-231 were treated with either HUVEC-CM or CTRL-CM for 48 h. Cells were trypsinized and reseeded into 96-well Seahorse plates in their respective media at 20 000 cells per well. After 24 h, Seahorse assays were performed following manufacturer instructions, with cells cultured in Seahorse XF DMEM during the assay. Relevant metabolic values (glycolysis and oxygen consumption rate) from each assay were calculated using template worksheets provided by Agilent. After completion of assays, DNA was extracted from each well using Caron's Buffer [25 mM Tris-HCl, 0.4 M NaCl, 0.5% (w/v) sodium dodecylsulfate]. Total DNA content per well was measured using the fluorometric Quantifluor dsDNA Assay (VWR) and used for normalization.

### Metabolomics and flux balance analysis

To obtain metabolite measurements to construct the FBA model, MDA-MB-231 were seeded in 24-well plates at 15 000 cells per well. 24 h after seeding, media was changed to either HUVEC-CM or CTRL-CM. Media was collected 0, 24, and 48 h after media change and stored at −80 °C before further analysis. Glucose concentration was measured using a GlucCell Glucose Monitoring System (CESCO Bioengineering), and lactate concentration was obtained using a colorimetric lactate assay kit (Sigma). Targeted metabolomics determining differences in the concentration of 20 amino acids was performed by the Proteomics and Metabolomics Core Facility at the Meyer Cancer Center of Weill Cornell Medicine using liquid chromatography-mass spectrometry (LC-MS). Briefly, stable isotope labeled amino acids (Cambridge Isotope Laboratories) were spiked into samples before samples were extracted using pre-chilled 80% methanol (−80 °C). The extract was dried with a Speedvac, and redissolved in HPLC (high-performance liquid chromatography) grade water before it was applied to the hydrophilic interaction chromatography LC-MS. Metabolites were measured on a Q Exactive Orbitrap mass spectrometer (Thermo Scientific), which was coupled to a Vanquish UPLC system (Thermo Scientific) via an Ion Max ion source with a HESI II probe (Thermo Scientific). A Sequant ZIC-pHILIC column (2.1 mm i.d. × 150 mm, particle size of 5 *μ*m, Millipore Sigma) was used for separation of metabolites. A 2.1 × 20 mm^2^ guard column with the same packing material was used for protection of the analytical column. Flow rate was set at 150 *μ*l/min. Buffers consisted of 100% acetonitrile for mobile phase A, and 0.1% NH_4_OH/20 mM CH_3_COONH_4_ in water for mobile phase B. The chromatographic gradient ran from 85% to 30% A in 20 min followed by a wash with 30% A and re-equilibration at 85% A. The Q Exactive was operated in full scan, polarity-switching mode with the following parameters: the spray voltage 3.0 kV, the heated capillary temperature 300 °C, the HESI probe temperature 350 °C, the sheath gas flow 40 units, and the auxiliary gas flow 15 units. MS data acquisition was performed in the m/z range of 70–1000, with 70 000 resolution (at 200 m/z). The automatic gain control (AGC) target was 1 × 10^6^ and the maximum injection time was 250 ms. The MS data were processed using XCalibur 4.1 (Thermo Scientific) to obtain the metabolite signal intensities. Quantitation was performed by comparing the signal intensities of the unlabeled amino acids with those of the labeled amino acids. All metabolic measurements were normalized to cell number and a blank solution of either HUVEC-CM or CTRL-CM.

Extracellular metabolomics data for amino acids, lactate, and glucose were used to constrain a flux balance analysis (FBA) model by imposing bounds on allowable exchange fluxes. To ensure convergence of the underlying linear programming problem solved by FBA, the simulations omitted constraints on the amino acids glycine, leucine, lysine, phenylalanine, threonine, tryptophan, tyrosine, and valine. However, the exchange fluxes for the remaining 12 amino acids, including the key amino acids glutamine and glutamate, were constrained in the simulations for all cases considered. In addition, the growth rate and O_2_ consumption fluxes were constrained by cell counts and Seahorse data, respectively. The model used in this study was implemented in the Julia programming language,^75^ where the linear programming problem was solved using the GNU Linear Programming Kit (GLPK) package (https://www.gnu.org/software/glpk/). The stoichiometric matrix and metabolic growth requirements were derived from a previously developed Core Cancer model.^27^ The objective function was set to maximize hyaluronic acid production, which was shown in a previous study to be a viable objective function to define the phenotype of interest;^38^ N = 1000 simulations were performed for each condition.

### Immunofluorescence

Cells in 2D culture were fixed in 4% paraformaldehyde (PFA) for 15 min. Fixed cultures were rinsed with PBS before permeabilization with 0.1% Triton X-100 and blocking with 2% BSA for 15 min and 1 h, respectively. Cultures were then incubated with anti-phospho Y397 FAK (pFAK; 1:100, raised in rabbit, clone EP2160Y) (Abcam) overnight at 4 °C. After PBS washing, cultures were incubated with goat anti-rabbit AlexaFluor-488 (ThermoFisher), AlexaFluor-568 Phalloidin (ThermoFisher), and DAPI (2.5 *μ*g/ml, ThermoFisher) for 1 h at room temperature. Stained cultures were washed with PBS and stored at 4 °C until imaging.

Cells in 3D microfluidic cultures were fixed in 4% PFA for 20 min by adding PFA to all media reservoirs. Fixed cultures were rinsed by replacing PFA with PBS before permeabilizing with 0.1% Triton X-100 for 15 min followed by blocking with 2% BSA for 1 h at room temperature. Primary staining with anti-CD31 (Sigma, 1:100, raised in mouse, clone WM-59) or with anti-pFAK (1:100) was performed overnight at 4 °C. Afterwards, cultures were rinsed with PBS overnight at 4 °C. Secondary staining with goat anti-rabbit Alexafluor-488, Alexafluor-568 Phalloidin, goat anti-mouse Alexafluor-647 (ThermoFisher), and DAPI occurred for 2 h at room temperature. Stained cultures were washed with PBS and allowed to rinse overnight at 4 °C before imaging.

### Confocal microscopy and image analysis

Images were acquired using a Zeiss LSM880 confocal microscope unless noted otherwise. For 2D experiments, a 32×/0.85 C-Achroplan objective with a 1.6× optical zoom was used. For 3D microfluidic cultures, a 10×/0.45 C-Aprochromat objective with a 0.6× optical zoom was used. Higher resolution images of microfluidic devices were obtained using the aforementioned 32× objective with a 1.6× zoom.

Custom ImageJ scripts were used for image analysis. Briefly, for 2D analysis, the maximum intensity projection (MIP) of Z-stack images was used to segment cells based on their F-actin intensity. Shape descriptors and the number of pFAK positive adhesions were obtained on a per cell basis. For invasion analysis in 3D microfluidic devices, the MIP of Z-stack images was used to segment cells based on their DAPI-stained cell nuclei. X-positions of each nucleus were obtained and averaged to determine invasion distances for the different experimental conditions. Invasion distance was normalized to the appropriate control in each set of replicates per experiment, and data were pooled together across three device replicates per condition.

To create color-coded morphology maps, a 20 slice (100 *μ*m) projection was created around the midpoint of the tumor channel. A Gaussian blur was performed, and the cells were segmented based on F-actin. After shape descriptors were obtained using built-in ImageJ functions, the ROI color coder ImageJ plugin was used to color segmented cells according to their aspect ratio.

To measure collagen contraction around individual cells, the ratio of collagen intensity in a 10 *μ*m region adjacent to cells (peripheral collagen) relative to bulk collagen (non-peripheral) was obtained using a custom ImageJ script. Briefly, confocal projections containing phalloidin stained cells and confocal reflectance imaging within devices were processed using the tubeness plugin to isolate collagen fiber signal. The f-actin signal was subsequently thresholded using Otsu's method to determine cell boundaries. A 10 *μ*m region around each cell boundary was created and confocal collagen reflectance intensity was measured in this peripheral region and averaged through each plane. The collagen confocal reflectance intensity was then measured in non-peripheral regions, and the ratio between the peripheral intensity and the non-peripheral intensity was calculated.

### FLIM analysis

For FLIM analysis, a Zeiss LSM880 confocal microscope coupled to a Spectra-Physics Insight DeepSee tunable femtosecond laser was used for FLIM image acquisition of NADH autofluorescence. Excitation was at 730 nm (∼100 fs pulse width) delivered by a 32×/0.85 C-Achroplan water immersion objective lens. Fluorescence was detected using a GaAsP PMT in the LSM880 non-descanned detector (NDD) unit through a 440/80 nm emission filter and 680 short pass filter (Chroma Technology, VT). Fluorescence lifetime images were acquired using time-correlated single photon counting of the NDD detector with a SPC-830 TCSPC card (Becker and Hickl, GMBL) in a separate computer. FLIM images (128 × 128 pixels) were integrated for 90 s by the SPC-830 card. The LSM880 scan speed was 2.2 *μ*m/*μ*s and laser power at the sample was 8.4 mW. Decay curves at each pixel were fit to a two-component multi-exponential model using the Becker & Hickl SPCImage program, and lifetime values and intensity were exported from SPCImage and further analyzed using a custom ImageJ script.

### Transwell invasion analysis

To supplement invasion studies in microfluidic devices, transwell migration experiments were performed. HUVECs were seeded at 250 000 cells per well in EGM-2 into a 24-well plate and allowed to attach overnight. 8 *μ*m pore-size transwell inserts (Corning) were coated with 50 *μ*g/ml rat-tail collagen type I for 1 h at 37 °C before washing with PBS. MDA-MB-231, HAS2-MCF10A, or MCF7 were seeded onto transwell inserts at 50 000 cells/mL. MDA-MB-231 and MCF7 were seeded in serum-free DMEM. HAS2-MCF10A were seeded in DMEM/F12 supplemented with 10 *μ*g/ml insulin, 0.5 *μ*g/ml hydrocortisone, 100 ng/ml cholera toxin, and 1 *μ*g/ml doxycycline. Transwells were cultured for 18 h (MDA-MB-231 and HAS2-MCF10A) or 48 h (MCF7) before fixation, and the number of cells that migrated through the transwell membrane were counted per field of view.

### Statistical analysis

All experiments were performed with at least three independent biological replicates unless otherwise noted. Pairwise comparisons were conducted using a Student's t-test with Welch's correction unless otherwise noted. Multiple comparisons were evaluated with a one-way ANOVA using Sidak's correction for multiple comparison. Results were considered statistically significant with a p-value less than 0.05. Unless otherwise noted, all data points are plotted as mean ± standard deviation. All statistical analysis was performed using GraphPad Prism v9.3 or R.

## SUPPLEMENTARY MATERIAL

See the supplementary material for additional characterization of tumor cells treated with HUVEC-CM, the effect of HUVEC-CM on tumor cell stemness, images of tumor-only invasion in microfluidic devices with metabolic inhibition, microfluidic device fabrication scheme and dimensions, and the full set of FBA results.

## Data Availability

The data that support the findings of this study are available from the corresponding author upon reasonable request.

## References

[1] S. Valastyan and R. A. Weinberg , “ Tumor metastasis: Molecular insights and evolving paradigms,” Cell 147, 275–292 (2011). 10.1016/j.cell.2011.09.024 22000009 PMC3261217

[2] D. X. Nguyen , P. D. Bos , and J. Massagué , “ Metastasis: From dissemination to organ-specific colonization,” Nat. Rev. Cancer 9, 274–284 (2009). 10.1038/nrc2622 19308067

[3] M. J. Oudin and V. M. Weaver , “ Physical and chemical gradients in the tumor microenvironment regulate tumor cell invasion, migration, and metastasis,” Cold Spring Harbor. Symp. Quant. Biol 81, 189–205 (2016). 10.1101/sqb.2016.81.030817 28424337

[4] K. R. Levental , H. Yu , L. Kass , J. N. Lakins , M. Egeblad , J. T. Erler , S. F. T. Fong , K. Csiszar , A. Giaccia , W. Weninger , M. Yamauchi , D. L. Gasser , and V. M. Weaver , “ Matrix crosslinking forces tumor progression by enhancing integrin signaling,” Cell 139, 891–906 (2009). 10.1016/j.cell.2009.10.027 19931152 PMC2788004

[5] T. Dean , N. T. Li , J. L. Cadavid , L. Ailles , and A. P. McGuigan , “ A TRACER culture invasion assay to probe the impact of cancer associated fibroblasts on head and neck squamous cell carcinoma cell invasiveness,” Biomater. Sci. 8, 3078–3094 (2020). 10.1039/C9BM02017A 32347842

[6] B.-Z. Qian and J. W. Pollard , “ Macrophage diversity enhances tumor progression and metastasis,” Cell 141, 39–51 (2010). 10.1016/j.cell.2010.03.014 20371344 PMC4994190

[7] Y. Y. Wang , C. Attané , D. Milhas , B. Dirat , S. Dauvillier , A. Guerard , J. Gilhodes , I. Lazar , N. Alet , V. Laurent , S. Le Gonidec , D. Biard , C. Hervé , F. Bost , G. S. Ren , F. Bono , G. Escourrou , M. Prentki , L. Nieto , P. Valet , and C. Muller , “ Mammary adipocytes stimulate breast cancer invasion through metabolic remodeling of tumor cells,” J. Clin. Invest. 2, e87489 (2017). 10.1172/jci.insight.87489 PMC5313068 28239646

[8] B. R. Seo , P. Bhardwaj , S. Choi , J. Gonzalez , R. C. Andresen Eguiluz , K. Wang , S. Mohanan , P. G. Morris , B. Du , X. K. Zhou , L. T. Vahdat , A. Verma , O. Elemento , C. A. Hudis , R. M. Williams , D. Gourdon , A. J. Dannenberg , and C. Fischbach , “ Obesity-dependent changes in interstitial ECM mechanics promote breast tumorigenesis,” Sci. Transl. Med. 7, 301ra130 (2015). 10.1126/scitranslmed.3010467 PMC4837896 26290412

[9] D. Ghosh , C. Mejia Pena , N. Quach , B. Xuan , A. H. Lee , and M. R. Dawson , “ Senescent mesenchymal stem cells remodel extracellular matrix driving breast cancer cells to a more-invasive phenotype,” J. Cell Sci. 133, jcs232470 (2020). 10.1242/jcs.232470 31932504 PMC6983709

[10] J. M. Szulczewski , D. R. Inman , M. Proestaki , J. Notbohm , B. M. Burkel , and S. M. Ponik , “ Directional cues in the tumor microenvironment due to cell contraction against aligned collagen fibers,” Acta Biomater. 129, 96–109 (2021). 10.1016/j.actbio.2021.04.053 33965625 PMC8848478

[11] K. Zhang , C. A. Corsa , S. M. Ponik , J. L. Prior , D. Piwnica-Worms , K. W. Eliceiri , P. J. Keely , and G. D. Longmore , “ The collagen receptor discoidin domain receptor 2 stabilizes SNAIL1 to facilitate breast cancer metastasis,” Nat. Cell Biol. 15, 677–687 (2013). 10.1038/ncb2743 23644467 PMC3794710

[12] M. W. Conklin , J. C. Eickhoff , K. M. Riching , C. A. Pehlke , K. W. Eliceiri , P. P. Provenzano , A. Friedl , and P. J. Keely , “ Aligned collagen is a prognostic signature for survival in human breast carcinoma,” Am. J. Pathol. 178, 1221–1232 (2011). 10.1016/j.ajpath.2010.11.076 21356373 PMC3070581

[13] W. Li , M. Khan , S. Mao , S. Feng , and J.-M. Lin , “ Advances in tumor-endothelial cells co-culture and interaction on microfluidics,” J. Pharm. Anal. 8, 210–218 (2018). 10.1016/j.jpha.2018.07.005 30140484 PMC6104288

[14] M. L. Tan , L. Ling , and C. Fischbach , “ Engineering strategies to capture the biological and biophysical tumor microenvironment *in vitro*,” Adv. Drug Delivery Rev. 176, 113852 (2021). 10.1016/j.addr.2021.113852 PMC8440401 34197895

[15] J. Lu , X. Ye , F. Fan , L. Xia , R. Bhattacharya , S. Bellister , F. Tozzi , E. Sceusi , Y. Zhou , I. Tachibana , D. M. Maru , D. H. Hawke , J. Rak , S. A. Mani , P. Zweidler-McKay , and L. M. Ellis , “ Endothelial cells promote the colorectal cancer stem cell phenotype through a soluble form of Jagged-1,” Cancer Cell 23, 171–185 (2013). 10.1016/j.ccr.2012.12.021 23375636 PMC3574187

[16] S. Krishnamurthy , K. A. Warner , Z. Dong , A. Imai , C. Nör , B. B. Ward , J. I. Helman , R. S. Taichman , E. L. Bellile , L. K. McCauley , P. J. Polverini , M. E. Prince , M. S. Wicha , and J. E. Nör , “ Endothelial interleukin-6 defines the tumorigenic potential of primary human cancer stem cells,” Stem Cells 32, 2845–2857 (2014). 10.1002/stem.1793 25078284 PMC4198458

[17] R. J. J. De Berardinis , N. S. S. Chandel , R. J. DeBerardinis , N. S. S. Chandel , R. J. J. De Berardinis , and N. S. S. Chandel , “ Fundamentals of cancer metabolism,” Sci. Adv. 2, e1600200 (2016). 10.1126/sciadv.1600200 27386546 PMC4928883

[18] J. C. García-Cañaveras , L. Chen , J. D. Rabinowitz , and J. C. García-ca , “ The tumor metabolic microenvironment: Lessons from lactate,” Cancer Res. 79, 3155–3163 (2019). 10.1158/0008-5472.CAN-18-3726 31171526 PMC6606343

[19] S. Hui , J. M. Ghergurovich , R. J. Morscher , C. Jang , X. Teng , W. Lu , L. A. Esparza , T. Reya , L. Zhan , J. Yanxiang Guo , E. White , J. D. Rabinowitz , L. Zhan , J. Y. Guo , E. White , J. D. Rabinowitz , L. Zhan , J. Yanxiang Guo , E. White , J. D. Rabinowitz , L. Zhan , J. Y. Guo , E. White , and J. D. Rabinowitz , “ Glucose feeds the TCA cycle via circulating lactate,” Nature 551, 115–118 (2017). 10.1038/nature24057 29045397 PMC5898814

[20] C. T. Hensley , A. T. Wasti , and R. J. Deberardinis , “ Glutamine and cancer: Cell biology, physiology, and clinical opportunities,” J. Clin. Invest. 123, 3678–3684 (2013). 10.1172/JCI69600 23999442 PMC3754270

[21] W. G. Kaelin and P. J. Ratcliffe , “ Review oxygen sensing by metazoans: The central role of the HIF hydroxylase pathway,” Mol. Cell 30, 393–402 (2008). 10.1016/j.molcel.2008.04.009 18498744

[22] M. R. Zanotelli , Z. E. Goldblatt , J. P. Miller , F. Bordeleau , J. Li , J. A. VanderBurgh , M. C. Lampi , M. R. King , and C. A. Reinhart-King , “ Regulation of ATP utilization during metastatic cell migration by collagen architecture,” Mol. Biol. Cell 29, 1–9 (2018). 10.1091/mbc.E17-01-0041 29118073 PMC5746062

[23] B. Cunniff , A. J. McKenzie , N. H. Heintz , and A. K. Howe , “ AMPK activity regulates trafficking of mitochondria to the leading edge during cell migration and matrix invasion,” Mol. Biol. Cell 27, 2662–2674 (2016). 10.1091/mbc.e16-05-0286 27385336 PMC5007087

[24] W. J. Polacheck , M. L. Kutys , J. B. Tefft , and C. S. Chen , *Microfabricated Blood Vessels for Modeling the Vascular Transport Barrier* ( Springer, 2019). 10.1038/s41596-019-0144-8 PMC7046311 30953042

[25] D.-H. T. Nguyen , E. Lee , S. Alimperti , R. J. Norgard , A. Wong , J. J.-K. Lee , J. Eyckmans , B. Z. Stanger , and C. S. Chen , “ A biomimetic pancreatic cancer on-chip reveals endothelial ablation via ALK7 signaling,” Sci. Adv. 5, eaav6789 (2019). 10.1126/sciadv.aav6789 31489365 PMC6713506

[26] J. D. Orth , I. Thiele , and B. Ø. Palsson , “ What is flux balance analysis?,” Nat. Biotechnol. 28, 245–248 (2010). 10.1038/nbt.1614 20212490 PMC3108565

[27] D. C. Zielinski , N. Jamshidi , A. J. Corbett , A. Bordbar , A. Thomas , and B. O. Palsson , “ Systems biology analysis of drivers underlying hallmarks of cancer cell metabolism,” Sci. Rep. 7, 41241 (2017). 10.1038/srep41241 28120890 PMC5264163

[28] N. E. Lewis and A. M. Abdel-Haleem , “ The evolution of genome-scale models of cancer metabolism,” Front. Physiol. 4, 237 (2013). 10.3389/fphys.2013.00237 24027532 PMC3759783

[29] T. J. Kwak and E. Lee , “ Rapid multilayer microfabrication for modeling organotropic metastasis in breast cancer,” Biofabrication 13, 015002 (2020). 10.1088/1758-5090/abbd28 32998119

[30] L. L. Munn , “ Aberrant vascular architecture in tumors and its importance in drug-based therapies,” Drug Discovery Today 8, 396–403 (2003). 10.1016/S1359-6446(03)02686-2 12706657

[31] J. P. Baskaran , A. Weldy , J. Guarin , G. Munoz , P. H. Shpilker , M. Kotlik , N. Subbiah , A. Wishart , Y. Peng , M. A. Miller , L. Cowen , and M. J. Oudin , “ Cell shape, and not 2D migration, predicts extracellular matrix-driven 3D cell invasion in breast cancer,” APL Bioeng. 4, 026105 (2020). 10.1063/1.5143779 32455252 PMC7202897

[32] M. Hanada , K. Tanaka , Y. Matsumoto , F. Nakatani , R. Sakimura , T. Matsunobu , X. Li , T. Okada , T. Nakamura , M. Takasaki , and Y. Iwamoto , “ Focal adhesion kinase is activated in invading fibrosarcoma cells and regulates metastasis,” Clin. Exp. Metastasis 22, 485–494 (2005). 10.1007/s10585-005-3733-6 16320111

[33] S. I. Fraley , Y. Feng , R. Krishnamurthy , D.-H. Kim , A. Celedon , G. D. Longmore , and D. Wirtz , “ A distinctive role for focal adhesion proteins in three-dimensional cell motility,” Nat. Cell Biol. 12, 598–604 (2010). 10.1038/ncb2062 20473295 PMC3116660

[34] S. P. Carey , Z. E. Goldblatt , K. E. Martin , B. Romero , R. M. Williams , and C. A. Reinhart-King , “ Local extracellular matrix alignment directs cellular protrusion dynamics and migration through Rac1 and FAK,” Integr. Biol. 8, 821–835 (2016). 10.1039/C6IB00030D PMC4980151 27384462

[35] J. T. Sharick , P. F. Favreau , A. A. Gillette , S. M. Sdao , M. J. Merrins , and M. C. Skala , “ Protein-bound NAD(P)H lifetime is sensitive to multiple fates of glucose carbon,” Sci. Rep. 8, 5456 (2018). 10.1038/s41598-018-23691-x 29615678 PMC5883019

[36] J. T. Sharick , C. M. Walsh , C. M. Sprackling , C. A. Pasch , D. L. Pham , K. Esbona , A. Choudhary , R. Garcia-Valera , M. E. Burkard , S. M. McGregor , K. A. Matkowskyj , A. A. Parikh , I. M. Meszoely , M. C. Kelley , S. Tsai , D. A. Deming , and M. C. Skala , “ Metabolic heterogeneity in patient tumor-derived organoids by primary site and drug treatment,” Front. Oncol. 10, 553 (2020). 10.3389/fonc.2020.00553 32500020 PMC7242740

[37] P. M. Schaefer , S. Kalinina , A. Rueck , C. A. F. von Arnim , and B. von Einem , “ NADH autofluorescence—A marker on its way to boost bioenergetic research,” Cytometry, Part A 95, 34–46 (2019). 10.1002/cyto.a.23597 30211978

[38] A. A. Shimpi , M. L. Tan , M. Vilkhovoy , D. Dai , L. D. M. Roberts , J. C. H. Kuo , L. Huang , J. D. Varner , M. Paszek , and C. Fischbach , “ Convergent approaches to delineate the metabolic regulation of tumor invasion by hyaluronic acid biosynthesis,” Adv. Healthcare Mater. 12, 2202224 (2022). 10.1002/adhm.202202224 PMC10238572 36479976

[39] A. Plaitakis , E. Kalef-Ezra , D. Kotzamani , I. Zaganas , and C. Spanaki , “ The glutamate dehydrogenase pathway and its roles in cell and tissue biology in health and disease,” Biology 6, 11 (2017). 10.3390/biology6010011 28208702 PMC5372004

[40] L. Tretter and V. Adam-Vizi , “ Alpha-ketoglutarate dehydrogenase: A target and generator of oxidative stress,” Philos. Trans. R. Soc., B 360, 2335–2345 (2005). 10.1098/rstb.2005.1764 PMC1569585 16321804

[41] T. Wang , F. Ma , and H. Qian , “ Defueling the cancer: ATP synthase as an emerging target in cancer therapy,” Mol. Ther.-Oncolytics 23, 82–95 (2021). 10.1016/j.omto.2021.08.015 34703878 PMC8517097

[42] I. K. Zervantonakis , S. K. Hughes-Alford , J. L. Charest , J. S. Condeelis , F. B. Gertler , and R. D. Kamm , “ Three-dimensional microfluidic model for tumor cell intravasation and endothelial barrier function,” Proc. Natl. Acad. Sci. 109, 13515–13520 (2012). 10.1073/pnas.1210182109 22869695 PMC3427099

[43] M.-E. Brett , H. E. Bomberger , G. R. Doak , M. A. Price , J. B. McCarthy , and D. K. Wood , “ *In vitro* elucidation of the role of pericellular matrix in metastatic extravasation and invasion of breast carcinoma cells,” Integr. Biol. 10, 242–252 (2018). 10.1039/C7IB00173H PMC6556113 29623978

[44] S. Kim , H. J. Kim , and N. L. Jeon , “ Biological applications of microfluidic gradient devices,” Integr. Biol. 2, 584 (2010). 10.1039/c0ib00055h 20957276

[45] J. S. Jeon , S. Bersini , M. Gilardi , G. Dubini , J. L. Charest , M. Moretti , and R. D. Kamm , “ Human 3D vascularized organotypic microfluidic assays to study breast cancer cell extravasation,” Proc. Natl. Acad. Sci. 112, 214–219 (2015). 10.1073/pnas.1417115112 25524628 PMC4291627

[46] S. Nagaraju , D. Truong , G. Mouneimne , and M. Nikkhah , “ Microfluidic tumor-vascular model to study breast cancer cell invasion and intravasation,” Adv. Healthcare Mater. 7, 1701257 (2018). 10.1002/adhm.201701257 29334196

[47] J. M. Ayuso , A. Gillette , K. Lugo-Cintrón , S. Acevedo-Acevedo , I. Gomez , M. Morgan , T. Heaster , K. B. Wisinski , S. P. Palecek , M. C. Skala , and D. J. Beebe , “ Organotypic microfluidic breast cancer model reveals starvation-induced spatial-temporal metabolic adaptations,” EBioMedicine 37, 144–157 (2018). 10.1016/j.ebiom.2018.10.046 30482722 PMC6284542

[48] J. M. Ayuso , M. Virumbrales-Munoz , P. H. McMinn , S. Rehman , I. Gomez , M. R. Karim , R. Trusttchel , K. B. Wisinski , D. J. Beebe , M. C. Skala , D. M. R. Karim , R. Trusttchel , K. B. Wisinski , J. Beebe , M. C. Skala , M. R. Karim , R. Trusttchel , K. B. Wisinski , D. J. Beebe , and M. C. Skala , “ Tumor-on-a-chip: A microfluidic model to study cell response to environmental gradients,” Lab Chip 19, 3461–3471 (2019). 10.1039/C9LC00270G 31506657 PMC6785375

[49] C. A. Lyssiotis and A. C. Kimmelman , “ Metabolic interactions in the tumor microenvironment,” Trends Cell Biol. 27, 863–875 (2017). 10.1016/j.tcb.2017.06.003 28734735 PMC5814137

[50] N. N. Pavlova and C. B. Thompson , “ The emerging hallmarks of cancer metabolism,” Cell Metab. 23, 27–47 (2016). 10.1016/j.cmet.2015.12.006 26771115 PMC4715268

[51] A. Halama , B. S. Guerrouahen , J. Pasquier , N. J. Satheesh , K. Suhre , and A. Rafii , “ Nesting of colon and ovarian cancer cells in the endothelial niche is associated with alterations in glycan and lipid metabolism,” Sci. Rep. 7, 39999 (2017). 10.1038/srep39999 28051182 PMC5209689

[52] M. Rossi , P. Altea-Manzano , M. Demicco , G. Doglioni , L. Bornes , M. Fukano , A. Vandekeere , A. M. Cuadros , J. Fernández-García , C. Riera-Domingo , C. Jauset , M. Planque , H. F. Alkan , D. Nittner , D. Zuo , L. A. Broadfield , S. Parik , A. A. Pane , F. Rizzollo , G. Rinaldi , T. Zhang , S. T. Teoh , A. B. Aurora , P. Karras , I. Vermeire , D. Broekaert , J. Van Elsen , M. M. L. Knott , M. F. Orth , S. Demeyer , G. Eelen , L. E. Dobrolecki , A. Bassez , T. Van Brussel , K. Sotlar , M. T. Lewis , H. Bartsch , M. Wuhrer , P. Van Veelen , P. Carmeliet , J. Cools , S. J. Morrison , J. Marine , D. Lambrechts , M. Mazzone , G. J. Hannon , S. Y. Lunt , T. G. P. Grünewald , M. Park , J. Van Rheenen , and S. Fendt , “ PHGDH heterogeneity potentiates cancer cell dissemination and metastasis,” Nature 605, 747–753 (2022). 10.1038/s41586-022-04758-2 35585241 PMC9888363

[53] S. S. Pinho and C. A. Reis , “ Glycosylation in cancer: Mechanisms and clinical implications,” Nat. Rev. Cancer 15, 540–555 (2015). 10.1038/nrc3982 26289314

[54] G.-N. Yan , L. Yang , Y. Lv , Y. Shi , L.-L. Shen , X.-H. Yao , Q.-N. Guo , P. Zhang , Y.-H. Cui , X. Zhang , X.-W. Bian , and D.-Y. Guo , “ Endothelial cells promote stem-like phenotype of glioma cells,” J. Pathol. 234, 11–22 (2014). 10.1002/path.4349 24604164 PMC4260128

[55] Z. Cao , J. M. Scandura , G. G. Inghirami , K. Shido , B.-S. Sen Ding , and S. Rafii , “ Molecular checkpoint decisions made by subverted vascular niche transform indolent tumor cells into chemoresistant cancer stem cells,” Cancer Cell 31, 110–126 (2017). 10.1016/j.ccell.2016.11.010 27989801 PMC5497495

[56] V. Snyder , T. C. Reed-Newman , L. Arnold , S. M. Thomas , S. Anant , L. Freire-de-lima , S. M. Thomas , V. Snyder , T. C. Reed-Newman , L. Arnold , S. M. Thomas , and S. Anant , “ Cancer stem cell metabolism and potential therapeutic targets,” Front. Oncol. 8, 203(2018). 10.3389/fonc.2018.00203 29922594 PMC5996058

[57] C. Chokchaitaweesuk , T. Kobayashi , T. Izumikawa , and N. Itano , “ Enhanced hexosamine metabolism drives metabolic and signaling networks involving hyaluronan production and *O*-GlcNAcylation to exacerbate breast cancer,” Cell Death Dis. 10, 803 (2019). 10.1038/s41419-019-2034-y 31645543 PMC6811536

[58] E.-C. Hsu , S. K. Kulp , H.-L. Huang , H.-J. Tu , S. B. Salunke , N. J. Sullivan , D. Sun , M. S. Wicha , C. L. Shapiro , and C.-S. Chen , “ Function of integrin-linked kinase in modulating the stemness of IL-6-abundant breast cancer cells by regulating γ-secretase-mediated Notch1 activation in caveolae,” Neoplasia 17, 497–508 (2015). 10.1016/j.neo.2015.06.001 26152358 PMC4719004

[59] C. M. Ghajar , H. Peinado , H. Mori , I. R. Matei , K. J. Evason , H. Brazier , D. Almeida , A. Koller , K. A. Hajjar , D. Y. R. Stainier , E. I. Chen , D. Lyden , and M. J. Bissell , “ The perivascular niche regulates breast tumour dormancy,” Nat. Cell Biol. 15, 807–817 (2013). 10.1038/ncb2767 23728425 PMC3826912

[60] G. S. Offeddu , C. Hajal , C. R. Foley , Z. Wan , L. Ibrahim , M. F. Coughlin , and R. D. Kamm , “ The cancer glycocalyx mediates intravascular adhesion and extravasation during metastatic dissemination,” Commun. Biol 4, 255 (2021). 10.1038/s42003-021-01774-2 33637851 PMC7910477

[61] P. Friedl and S. Alexander , “ Cancer invasion and the microenvironment: Plasticity and reciprocity,” Cell 147, 992–1009 (2011). 10.1016/j.cell.2011.11.016 22118458

[62] P. Friedl and K. Wolf , “ Plasticity of cell migration: A multiscale tuning model,” J. Cell Biol. 188, 11–19 (2010). 10.1083/jcb.200909003 19951899 PMC2812848

[63] E. Cukierman , R. Pankov , D. R. Stevens , and K. M. Yamada , “ Taking cell-matrix adhesions to the third dimension,” Science 294, 1708–1713 (2001). 10.1126/science.1064829 11721053

[64] W. D. Leineweber and S. I. Fraley , “ Adhesion tunes speed and persistence by coordinating protrusions and extracellular matrix remodeling,” Dev. Cell 58, 1414 (2023). 10.1016/j.devcel.2023.05.013 37321214 PMC10527808

[65] H. Yi , G. Talmon , and J. Wang , “ Glutamate in cancers: From metabolism to signaling,” J. Biomed. Res. 34, 260–270 (2020). 10.7555/JBR.34.20190037 PMC7386414 32594024

[66] A. R. Mullen , Z. Hu , X. Shi , L. Jiang , L. K. Boroughs , Z. Kovacs , R. Boriack , D. Rakheja , L. B. Sullivan , W. M. Linehan , N. S. Chandel , and R. J. DeBerardinis , “ Oxidation of alpha-ketoglutarate is required for reductive carboxylation in cancer cells with mitochondrial defects,” Cell Rep. 7, 1679–1690 (2014). 10.1016/j.celrep.2014.04.037 24857658 PMC4057960

[67] J. Pasquier , P. Ghiabi , L. Chouchane , K. Razzouk , S. Rafii , and A. Rafii , “ Angiocrine endothelium: From physiology to cancer,” J. Transl. Med. 18, 52 (2020). 10.1186/s12967-020-02244-9 32014047 PMC6998193

[68] P. Carmeliet and R. K. Jain , “ Molecular mechanisms and clinical applications of angiogenesis,” Nature 473, 298–307 (2011). 10.1038/nature10144 21593862 PMC4049445

[69] M. Murgai , W. Ju , M. Eason , J. Kline , D. W. Beury , S. Kaczanowska , M. M. Miettinen , M. Kruhlak , H. Lei , J. F. Shern , O. A. Cherepanova , G. K. Owens , and R. N. Kaplan , “ KLF4-dependent perivascular cell plasticity mediates pre-metastatic niche formation and metastasis,” Nat. Med. 23, 1176–1190 (2017). 10.1038/nm.4400 28920957 PMC5724390

[70] L. Xiao , J. V. McCann , and A. C. Dudley , “ Isolation and culture expansion of tumor-specific endothelial cells,” J. Vis. Exp. 2015, e53072. 10.3791/53072 PMC4692661 26554446

[71] K. Taguchi , T. Onoe , T. Yoshida , Y. Yamashita , K. Taniyama , and H. Ohdan , “ Isolation of tumor endothelial cells from murine cancer,” J. Immunol. Methods 464, 105–113 (2019). 10.1016/j.jim.2018.11.005 30395818

[72] M. L. Kutys , W. J. Polacheck , M. K. Welch , K. A. Gagnon , T. Koorman , S. Kim , L. Li , A. I. McClatchey , and C. S. Chen , “ Uncovering mutation-specific morphogenic phenotypes and paracrine-mediated vessel dysfunction in a biomimetic vascularized mammary duct platform,” Nat. Commun. 11, 3377 (2020). 10.1038/s41467-020-17102-x 32632100 PMC7338408

[73] P. S. Thiagarajan , M. Hitomi , J. S. Hale , A. G. Alvarado , B. Otvos , M. Sinyuk , K. Stoltz , A. Wiechert , E. Mulkearns-Hubert , A. M. Jarrar , Q. Zheng , D. Thomas , T. T. Egelhoff , J. N. Rich , H. Liu , J. D. Lathia , and O. Reizes , “ Development of a fluorescent reporter system to delineate cancer stem cells in triple-negative breast cancer,” Stem Cells 33, 2114–2125 (2015). 10.1002/stem.2021 25827713 PMC4494654

[74] K. M. Chrobak , D. R. Potter , and J. Tien , “ Formation of perfused, functional microvascular tubes in vitro,” Microvasc. Res. 71, 185–196 (2006). 10.1016/j.mvr.2006.02.005 16600313

[75] J. Bezanson , A. Edelman , S. Karpinski , and V. B. Shah , “ Julia: A fresh approach to numerical computing,” SIAM Rev. 59, 65–98 (2017). 10.1137/141000671

